# Superdiversity and Disability: Social Changes for the Cohesion of Migrations in Europe

**DOI:** 10.3390/ijerph17186460

**Published:** 2020-09-04

**Authors:** Mª del Carmen Martín-Cano, Cristina Belén Sampedro-Palacios, Adrián Jesús Ricoy-Cano, Yolanda María De La Fuente-Robles

**Affiliations:** Social Work Department, University of Jaén, 23071 Jaén, Spain; mmcano@ujaen.es (M.d.C.M.-C.); cbsamped@ujaen.es (C.B.S.-P.); ymfuente@ujaen.es (Y.M.D.L.F.-R.)

**Keywords:** migrations, disability, superdiversity, social changes, social work

## Abstract

In recent years, international migration has changed considerably, improving our understanding of the diversity of migrants, something that until recently was viewed as a fixed pattern associated with the ethnic group in question. At the same time, in the international context, the importance and the need to recognize the rights of people with disabilities has grown. Therefore, the purpose of this paper is to provide a more detailed analysis of this phenomenon in Europe, from the perspective of superdiversity, which covers the different variables that come into play, as well as the responses to the diverse needs that are provided through the action protocols in host countries. To address the objective of this research, we present a critical review of the migration policies undertaken at the European level, methodologically approached using the causal inference model. Our findings show a lack of structure of social and professional intervention policies, at the international level, towards refugees with disabilities. We conclude by presenting a series of political guidelines that rely on scientific evidence to improve the lives of migrants with disabilities.

## 1. Introduction

Millions of people are continually forced to leave their home countries for various reasons. According to data from the UN Refugee Agency, UNHCR [1], in 2017, 68.5 million people were forced to flee their countries: of these, 4.4 million people emigrated to one of the EU-28 Member States [2]. It is estimated that a considerable number of them are people with disabilities, although very little information is available about them [3].

There are many reasons why people move to another country, sometimes voluntarily for professional or academic reasons, but there are many others who do so forced by a conflictive situation or catastrophe in their country of origin [4]. Similarly, there are many complexities involved in the phenomenon of migration, giving rise to what Vertovec defines as “superdiversity”, understood as “a dynamic interplay of variables among an increased number of new, small and scattered, multiple-origin, transnationally connected, socio-economically differentiated and legally stratified immigrants” [5]. In other words, the term superdiversity indicates that not only are there differences between the people of the host country and citizens of other nationalities, but that there are also differences between all immigrants in terms of their multiple origins, their socioeconomic differences, and different languages, etc. Put another way, Vertovec points out that we are talking about a diversification of diversity.

The growing notoriety of these social issues in the international arena leads to questions about the interrelations that occur between migration and disability. In keeping with Crenshaw’s [6] intersectionality theory, according to which each individual suffers oppression or holds privilege based on their membership in multiple social categories, if the population with disabilities and the migrant population are separately subject to political action because of the vulnerability of their situation, then being a migrant while having a disability must be approached through the interaction of the inequalities faced by the collective. Hence the need to study the characteristics of the migrant population with disabilities and the obstacles that must be overcome in different contexts so as to allow for the design of adequate planning to develop effective policies that address their needs [7]. 

It should be noted, as McAuliffe and Ruhs point out [8], that the International Organization for Migration recognizes disability as an element of vulnerability:

Vulnerable migrants are those who, even without meeting the requirements to receive protection under refugee frameworks, face a variety of situations in their home countries that endanger their lives or are subject to discrimination based on any reason (p. 159 [8]).

A politically effective response to the current migration crisis in Europe requires a greater understanding of the causes of migration. Inconsistencies in European statistics on irregular migration make this difficult. However, there are two key drivers for this phenomenon to occur. The first involves the combination of conflicts and political instability, and the second stems from the economic insecurity in the countries of origin, which seems to be the engine of migration [9].

Moreover, people seeking refuge in a foreign country often experience trauma and distress due to their uncertain residential and legal status. Previous research has identified how the relevant services and the creation of policies continue to be precarious in responding to their needs [10].

Under the generic paradigm of disability and immigration, it is necessary to understand common situations in which both realities are combined with everyday activities. Previous research has pointed out clarifying examples, such as the educational reality of migrant children with disabilities [11] or the employment situation [12]. All of them are necessary for a greater understanding of the phenomenon of migration and for improving the living conditions of migrants with disabilities.

The general objective of this study is to analyze the phenomenon of migration in Europe from the perspective of superdiversity, as well as the social responses that are currently offered through the action protocols in different host countries. In addition to this general objective, the following research questions are presented to provide a guide for the synthesis of information from the scientific literature:

**Research question 1:** Why is it necessary to speak of superdiversity in migratory movements today?

**Research question 2:** What are the implications of dual vulnerability when the condition of being a migrant and having a disability intersect in an individual?

**Research question 3:** What action protocols are being carried out to improve the coexistence of migrants with functional diversity in host countries?

To respond to the proposed general objective and the different research questions, we provide a critical review and analysis of legal, political and human rights documents, methodologically approached using the causal inference model.

## 2. Methodology

A review of the literature was conducted in the Web of Science, Scopus, PsycINFO ProQuest and PubMed databases, in the Google Scholar academic search engine, international web pages related to the field of research, and Legislation and Jurisprudence Databases.

The methodology applied for the development of this legal and human rights policy document is based on the Frankfort–Nachmias and Nachmias model of causal inferences [13].

Among the main components of the inference model, it is necessary to point out the strength of the causal approaches, since in them lies the identification of the social mechanisms that will explain the relationship between a cause and its corresponding effect. Being able to discover what the mechanism is through research also facilitates an articulated reconstruction of events. All this leads to an explanatory–causal model. Consequently, different theoretical perspectives are selected, considering the following operational path in the research design (data → information → evidence), collecting and assembling the evidence with data and information.

The ultimate goal is to create a structure capable of providing a comprehensive political analysis of migration and disability in Europe that is able to assemble significant evidence and thus provide the opportunity to propose solutions or mitigate the negative consequences of this phenomenon. 

## 3. Migration and Disability: A Challenge with No Answer

Migrations are among the main factors that contribute to growing diversity, a diversity that cannot be understood solely as the presence of multiple cultures in a society. Therefore, if disability and ethnicity are linked and we are able to talk about “superdiversity plus”, how does the world act in the face of superdiversity plus? The answer must be based on conviviality, understood as processes of cohabitation and interaction that turn multiculture into a common feature of social life. This is because conviviality [11] and superdiversity [5] are concepts that, when applied to certain realities, are intended to guide the daily management of said differences.

It is essential to link citizenship and human rights in contemporary societies in the context of migration and disability, but the issue becomes complicated in contexts of crisis and austerity, and as a result, the reduction of available services solidifies the idea of whether the person who is receiving said services “is one of us”. For all these reasons, we must rethink diversity by placing governance at the center of any progress, in a place closely linked to hyperdiversity and social cohesion [14].

Between 2015 and 2016, there was a massive influx of applicants for international protection in the European Union. That period was a turning point that triggered a change in European strategy that would create the necessary network to define new social policies and provide a solution to a huge list of unanswered needs.

In 2014, the number of first-time applicants for refugee status was 562,675 (women: 164,155). In 2015, this figure increased considerably to 1,257,035 (women: 344,390). In 2016, it fell slightly to 1,206,115 (women: 389,165) [15], falling further in 2017 to a figure similar to that of 2014 at 619,685 (women: 204,355), likely as a result of the agreed containment policies. Refugees, asylum seekers and other migrants with disabilities are not properly identified. This invisibility makes it difficult to plan the necessary support measures so that they can have equal access to services in reception centers [7]. In this era of “superdiversity” [4,5], the rights of individuals are a key concern.

In this sense, there is a set of regulatory, legal and human rights frameworks that support immigrants, both with and without disabilities. Migration and displacement are important issues for both policy-makers and human rights and development professionals [16]. In 2014, the United Nations presented the document “The economic, social and cultural rights of migrants in an irregular situation” [17,18], which lists fundamental rights, such as the right to health, the right to an adequate standard of living, including housing, water and sanitation, and food, the right to education, the right to social security, the right to work and the right to just and favorable working conditions.

More specifically, for people with disabilities, the Common European Asylum System (CEAS) defines the set of regulations at the European Union level that, since 2003, has addressed the issue of refugees with disabilities, including the following:

− Council Directive 2003/9/EC of 27 January 2003, laying down minimum standards for the reception of asylum seekers in the Member States, Article 17 of which specifies that “Member States shall take into account the specific situation of vulnerable persons such as minors, unaccompanied minors, disabled people, elderly people, pregnant women, single parents with minor children and persons who have been subjected to torture, rape or other serious forms of psychological, physical or sexual violence, in the national legislation implementing the provisions of Chapter II relating to material reception conditions and health care”.
− Council Directive 2005/85/EC of 1 December 2005 on minimum standards on procedures in Member States for granting or withdrawing refugee status, which guarantees access to fair and effective asylum procedures [19].
− Directive 2011/95/EU of the European Parliament and of the Council of 13 December 2011 on standards for the qualification of third-country nationals or stateless persons as beneficiaries of international protection, for a uniform status for refugees or for persons eligible for subsidiary protection, and for the content of the protection granted. This Directive states that it is appropriate to guarantee access to health care, both physical and mental health care, to beneficiaries of international protection [20] and clarifies in Article 30.2 that Member States shall provide adequate health care including, if necessary, the treatment of mental disorders, to beneficiaries of international protection who have special needs, such as pregnant women, disabled people, persons who have undergone torture, rape or other serious forms of psychological, physical or sexual violence or minors who have been victims of any form of abuse, neglect, exploitation, torture, cruel, inhuman or degrading treatment or who have suffered from armed conflict, under the same eligibility conditions as nationals of the Member State that granted them protection [21].
− Directive 2013/33/EU of the European Parliament and of the Council of 26 June 2013 laying down standards for the reception of applicants for international protection, Article 21 of which classifies refugees with a disability in the group of especially vulnerable people.

In the national law implementing this directive, Member States shall take into account the specific situation of vulnerable persons such as minors, unaccompanied minors, disabled people, elderly people, pregnant women, single parents with underage children, victims of human trafficking, persons with serious illnesses, persons with mental disorders and persons who have been subjected to torture, rape or other serious forms of psychological, physical or sexual violence, such as victims of female genital mutilation [22,23].

There are also international standards that specifically protect disabled refugees: for example, Article 11 of the Convention on the Rights of Persons with Disabilities of the United Nations states that parties shall take, in accordance with their obligations under international law (including international humanitarian law and international human rights law), all necessary measures to ensure the protection and safety of persons with disabilities in situations of risk, including situations of armed conflict, humanitarian emergencies and the occurrence of natural disasters.

On 19 September 2016, the General Assembly of the United Nations approved the New York Declaration for Refugees and Migrants [24], which established that the States will address:

In accordance with our obligations under international law, the special needs of all people in vulnerable situations who are travelling within large movements of refugees and migrants, including women at risk, children (especially those who are unaccompanied or separated from their families), members of ethnic and religious minorities, victims of violence, older persons, persons with disabilities, persons who are discriminated against on any basis, indigenous peoples, victims of human trafficking, and victims of exploitation and abuse in the context of the smuggling of migrants.

Specifically, they commit to using the registration process to identify specific assistance needs and protection arrangements, where possible, including but not exclusively for refugees with special protection concerns, such as women at risk, children, especially unaccompanied children and children separated from their families, child-headed and single-parent households, victims of trafficking, victims of trauma and survivors of sexual violence, as well as refugees with disabilities and older persons.

In response to the Committee’s recommendations, the European Parliament adopted the European Parliament Resolution of 7 July 2016 on the implementation of the United Nations Convention on the Rights of Persons with Disabilities, with special regard to the Concluding Observations of the United Nations Committee on the Rights of Persons with Disabilities (2015/2258 (INI)). The European Parliament [25]:

58. Recognizes that vulnerable members of society are further marginalized if they have a disability, and stresses that the EU institutions and the Member States should redouble their efforts to fully accommodate the provision of rights and services for all persons with disabilities, including stateless people, homeless people, refugees and asylum seekers and people belonging to minorities; underlines the need to mainstream disability in the EU’s migration and refugee policies;

59. Asks the Commission and the Council, in accordance with Article 11 of the Convention on the Rights of Persons with Disabilities (CRPD), when making proposals for resolving the refugee issue, for funding or for other support measures, to provide for special care for persons with disabilities.

Additionally, the diversity between the different Member States is not only considerable in terms of the number of applications submitted, but also in terms of public policies, the policies of the respective governments and the response of societies. However, the common denominator in every country is the deficiencies in the reception systems for this group, which pose a serious risk of vulnerability and exclusion. Care systems for international protection applicants and disabled and/or dependent refugees are often insufficient, and frequently caused by traumatic situations suffered in countries of origin or during their escape.

## 4. Non-Homogeneous Responses to Diverse Needs in Superdiversity

The notion of superdiversity encompasses changes in the multiple dimensions presented by migration standards. As Wessendorf [26] points out, it is configured as the prism through which to describe “an exceptional demographic situation characterized by the multiplication of social categories within specific localities” (p. 1287 [26]).

This term was first used by Vertovec [5] to describe the changing patterns observed in migration data in the United Kingdom, where not only had the number of people from different countries increased, so had their ethnicities, languages and religions.

It was observed that ethnic diversity alone is not enough by itself to describe the phenomenon of migration; rather, it is characterized by being a dynamic interaction between the different combinations of variables that come into play such as gender, age, generation, legal status, education, and others.

However, “many of those who use the term have referred only to more ethnicities rather than to the more complete original intention of the term to recognize multidimensional changes in migration patterns. This implies a worldwide diversification of migration channels, differentiation of legal states, divergent patterns of gender and age, and a variation in the human capital of migrants” [27].

In this sense, in a context where international migration has changed considerably, the idea of unique forms of diversity centered around a fixed pattern determined by the ethnicity of the migrants in question is now outdated. This phenomenon must be analyzed from the perspective of a multidimensional prism that spans the different variables that come into play. Obviously, between the different groups of emigrants and within each one of them, regardless of their origin, there are significant differences between generations, between women and men, as well as between people with different educational levels. Therefore, a change is needed in the analysis used, one that goes beyond the membership group to encompass the dynamic interaction between the different individual characteristics of each of its members, from a multidimensional prism [28] that goes beyond the limits of the group to consider variables that, until now, were ignored, such as functional diversity.

In order to consider the vulnerability of a migrant, their situation and individual needs must be thoroughly evaluated regardless of their predefined category, since what defines their potential vulnerability is the combination of both intrinsic and extrinsic characteristics and circumstances at a given time.

In Europe, community policies on migration have been posed within the framework of freedom, security and justice considerations, rather than that of the free movement of people, and have focused on limiting entry into community territory of citizens from third countries for professional purposes and on establishing effective borders against irregular immigration. Each Member State has imposed the function of safeguarding the borders of the European Union against uncontrolled migratory flows and ensuring the protection of all the territories of the States against illegal immigration.

The EU’s treatment of immigration has a dual role: to ensure the legal integration of the immigrant, placing the individual and their rights (especially minors and women) at the center; and to treat irregular immigration from the perspective of controlling migratory flows, protecting the internal labor market and assuring the gradual integration of immigrants into indigenous society. From this dual perspective, an attempt is made to design a coherent and integrated framework between national and European policies.

Among all immigrant groups, asylum seekers, refugees and other migrants with disabilities are not properly identified and do not have equal access to services in reception centers, and that is precisely where the problem begins. Additionally, the diversity between the different Member States is not only considerable in terms of the number of applications submitted, but also in terms of public policies, the policies of the respective governments and the response of societies. However, the common denominator in every country is deficiencies in reception systems that pose a serious risk of vulnerability and exclusion. Care systems for international protection applicants and refugees who are disabled and/or dependent are often insufficient, and frequently result from traumatic situations suffered in their countries of origin or during their escape.

Among the deficits identified are the problems in diagnosing the specific needs of people who are disabled and/or dependent, legal restrictions that prevent them from accessing regular care services, lack of accessibility in reception facilities, lack of employment offers, and insufficient cooperation between the systems responsible for receiving refugees and those that are tasked with caring for persons with disabilities.

The International Federation of Red Cross and Red Crescent Societies [29] believes that the best way to support migrants is by helping them be resilient throughout their journey. If they have that capacity, they can better address the risks and overcome the external crises associated with migration. While every aspect of resilience and recovery is important, at certain times of the journey, some aspects are more prominent than others.

## 5. Building True Citizenship: Global Solutions to the Needs of People with Disabilities

According to Human Rights Watch research [30], among the deficits identified are the problems with diagnosing the specific needs of people who are disabled and/or dependent, legal restrictions that prevent them from accessing regular care services, lack of accessibility in reception facilities, lack of employment offers, and insufficient cooperation between the systems responsible for receiving refugees and those that are tasked with caring for persons with disabilities.

The UN refugee agency (UNHCR) and international and local relief organizations working with refugee centers in Greece informed Human Rights Watch [30] that they have very few or no programs specifically designed to address the rights and needs of asylum seekers, refugees and other migrants with disabilities. Both asylum seekers and other migrants with disabilities face enormous difficulties obtaining basic services such as shelter, sanitation and medical care, and like other vulnerable migrants, they have limited access to mental health care. Based on research carried out in Greece between 2016 and 2017, Human Rights Watch concluded that in Greece, asylum seekers and refugees with disabilities are not properly identified, partly because the registration process is rushed and the staff lack proper training. Without an adequate understanding of the magnitude and needs, assistance agencies cannot respond effectively.

To end this dual discrimination, the EU should request information from its Member States on the execution of its programs to ensure that the projects they finance benefit people with disabilities and other groups at risk.

All this happens despite the fact that the various European Directives and international standards are unquestionable, urging Member States to take into account these and other especially vulnerable groups; and yet, compliance with them is usually the exception [31,32,33,34].

To lay the foundations for a new way forward to correct all these imbalances in the procedure, a hearing on “The situation of refugees and migrants with disabilities” was held in Brussels (2017). The objective of this hearing was to draw attention to this particularly vulnerable group of refugees and immigrants, trying to raise awareness of the rights and needs of people with disabilities through the international organizations that work with them. Most significantly, it was noted that European regulation (Directive 2003/9 of 27 January 2003, laying down minimum standards for the reception of asylum seekers in Member States) requires Member States to take into account the specific situation of vulnerable persons, especially in relation to reception conditions, individually assessing their particular needs, specifically those related to a disability. One of the problems highlighted is the lack of a homogeneous response by the Member States when it comes to offering protection to vulnerable people who come to Europe in search of asylum, so in many cases, the integration of migrants with disabilities, as well as their access to social rights, is still precarious.

Among the main conclusions of the meeting, we note the following [35]:

1. Article 11 of the Convention on the Rights of Persons with Disabilities, which requires participating States to take all necessary measures to ensure the protection and safety of persons with disabilities at risk, must be fully implemented.
2. It is necessary to have accurate data on the number of people with disabilities among refugees and migrants. To date, the records are either unavailable or unreliable.
3. Access to asylum applications must be guaranteed by adapting to people with disabilities.
4. A comprehensive approach to all basic rights (medical care, housing, education, etc.) needs to be taken, taking into account functional diversity.
5. Cooperation between the different organizations and institutions that work with refugees and people with disabilities.
6. We must advance the resettlement system and shorten the deadlines for family reunification in cases of vulnerability.
7. The capacities of local authorities need to be strengthened (pp. 67–68 [35]).

The Global Pact on Safe, Orderly and Regular Migration of 2018 [36], together with the International Convention on the Rights of Persons with Disabilities in 2006, both of the United Nations [37], are the main bases that establish the reality of migration and disability in Europe, adherence to which would imply undertaking actions aimed at protecting migrants with disabilities within a paradigm of superdiversity. However, since they are not binding regulations, States find shortcuts with which to manage both realities based on their own economic and intervention paradigm.

Although public actors play an important role both in making visible and in ensuring the protection of vulnerable groups, it is the different levels of intervention by the States that are unbalanced in terms of government actions. A lack of coordination in the creation and application of legislation is one of the reasons why migrants with disabilities are currently in a situation of potentially understandable extrinsic vulnerability [38].

## 6. Creation of New Protocols as a Tool for Coexistence

For decades, international migration has been one of the factors that has contributed the most to cultural diversity. However, when migratory studies focus on integration, their analysis of intercultural diversity is limited. That is why new paradigms have emerged—although still poorly implemented—with a more holistic vision, which include patterns of relationships, interactions and types of influences between immigrant and native residents. However, much remains to be done before the need for coexistence in global cities is fully conceived [39].

As Berisso and Giuliano [40] point out, the relationship between liberation and interculturality requires coexistence to prevail over competitiveness. This necessitates an educational process that promotes the eradication of the factors that exalt a dominant Western epistemology of humanity’s knowledge over that of other cultures.

Observing diversity from a sociological perspective makes it possible to accentuate the painful historical evidence that there is no diversity without power and asymmetry, but at the same time we must not lose sight of the fact that there is a daily component of the difference that passes through the subjective dynamics used in intercultural relationships [41].

In this regard, Amín [42] points out the weight that daily life holds in neighborhoods, workplaces, and public spaces, where historical, global and local processes intersect to make sense of living with diversity.

Given the bi-directionality that integration entails, the structural guidelines of the host society are essential to determine the possibilities of integrating immigrant groups. The characteristics of the labor market and the welfare model are therefore configured as determining elements, but the economic and demographic structure of each region must also be taken into account, as well as the institutional capacity to ensure adequate reception for those arriving from another territory [43].

In the comparative study that Crul [28] performed on diversity and assimilation in the European cities of Amsterdam, Stockholm and Berlin, he points out that “the theory of segmented assimilation maintains that some ethnic groups find themselves more frequently on a descending path, while others find themselves more frequently on an ascending path”. This issue is largely explained by the different forms of reception, as well as by the ethnicity and socio-economic peculiarities of the first generation (p. 63 [28]).

However, according to the author, in addition to the ethnic factor, in the case of Amsterdam, background and contextual factors also play a very important role. Regarding social mobility, the results of the study reflect a dual reality; on one hand, it presents an upward social mobility, in contrast to Berlin, where stagnant or downward mobility prevails. The theory of diversity as reformulated in the aforementioned study weighs the need to observe the discrepancies within groups related to differences in local and national contexts.

Therefore, it is unavoidable to design an action protocol from the praxis of social work for coexistence, governed by three fundamental principles—universality, active integration and intercultural coexistence—in order to offer a global response to the different problems of migrants. This will allow this heterogeneous collective to be freely and fully incorporated and to experience equal rights, duties, and opportunities, just like the rest of the host population.

## 7. From Observation to Intervention

As a consequence of the aforementioned facts, our knowledge of the incidence and specific cases involving disability in the group of applicants for international protection is quite poor. Most studies cover different forms of vulnerability and analyze the situation of refugee camps in neighboring countries, comparing them to those of the originating country [44].

An example of this are the reports of the Women’s Commission for Refugee Women [45], which describe the situation of refugees in camps located in urban areas in five developing countries and Syrian refugees in Lebanon. The reports analyze the lack of intervention protocols for the population of refugees with disabilities and the need for inventiveness in the face of the various situations they had to endure every day to respond to the situation [46]. Other examples are the studies of Roberts and Harris [47] and Ward, Amas and Lagnado [48], where they analyze the care given to refugees with a disability in the United Kingdom, re-highlighting the need for intervention design; similarly, Mirza and Heinemann [49] detail the situation in the U.S., in which they examine the suitability of existing services in the system to address the different needs of refugees with disabilities. They conclude that these refugees have limited access to resettlement resources due to their doubly vulnerable situation resulting from their status as both migrants and people with disabilities. In addition to concluding that, the main impediment to addressing the reality of refugees with disabilities is the lack of coordination between refugee systems and people with disabilities.

Several studies [50,51,52,53] conducted by Handicap International and other institutions detail the challenges facing refugee care, a situation that can be extrapolated to the different EU countries:

1. Deficit in diagnosing and identifying the care needs of refugees with a disability, which leads to a considerable waste of time and the transfer of these refugees to reception centers that do not have the resources to guarantee adequate treatment.
2. Legal restrictions that prevent refugees with a disability from accessing regular care services.
3. Lack of accessibility in the facilities (reception centers, language academies, institutions, etc.).
4. Lack of resources for language learning appropriate to the needs of people with a disability.
5. Lack of specific offers to promote the employability of refugees with a disability.
6. Ignorance of support structures for people with a disability by the refugees themselves.
7. Insufficient cooperation between the systems responsible for receiving refugees and caring for people with disabilities.
8. Excessive complexity and bureaucratization of the care systems for people with a disability.
9. Civil society initiatives for refugee support cannot handle the complexity of supporting refugees with a disability.
10. Lack of adapted surfaces and spaces that allow for the integration of refugees with a disability.

The European Commission has identified twelve main challenges, including immigration, as a reality that virtually all European societies have to face [54,55].

In the area of immigration policies, although they have often been framed in the context of national integration models, at present, human mobility is placing immigration at the center of local political agendas. Recent studies [56,57,58,59,60,61] focus attention at the local level, mainly the city. Cities are becoming increasingly active agents, drawing up their own agendas and developing specifically local political strategies to address the integration challenges of immigrants.

Thus, for example, policies for integrating immigrants belong to the local field of action for several reasons: municipalities are the appropriate administrative level to implement local policies, since the logic of municipal policies is different from that of the central states; and only municipal policy can mobilize local resources, both formal and informal [62].

In terms of immigration and integration, the perspective of municipalities is therefore radically different from that of central governments [63,64]. In fact, in this set of policies, municipalities have played a pioneering role, promoting integration policies, obviating the criticisms of the central government, since they cannot close their eyes to pressing and immediate immigration-related problems [65]. In some countries, such as the Netherlands and Germany [66], central governments have ended up adopting postulates and central instruments of municipal policies.

In short, the commitment to local policies to integrate immigrants implies a new approach to address and promote diversity, overcoming the traditional state model of multiculturalism and assimilationism [67]. This approach has been supported by international organizations and networks of transnational cities, like the Council of Europe, which founded the network of Intercultural Cities. This development clearly points to the relevance of horizontal relationships, from city to city, of local governments [56].

## 8. Discussion and Conclusions

The lack of data on migrants with disabilities requires us to tackle a hidden population without an adequate understanding of their magnitude and needs; as a result, the action of the public or private institutions tasked with guaranteeing their rights is not effective. In fact, although national and international standards are unquestionable when it comes to protecting these types of especially vulnerable situations, compliance with them is usually the exception. Therefore, we must commit to a rigorous application of the law in this regard in all countries.

Heterogeneity and deficiencies in the systems for receiving this group of people, who are at serious risk of vulnerability and exclusion, are a common denominator at the international level. The care systems for international protection applicants and refugees with functional diversity, regardless of their origin or cause of the migration process and/or escape, are particularly insufficient. There are minimal programs that do not adequately identify asylum seekers and refugees with disabilities, showing deficiencies in their records and in the training and preparation of the professionals involved.

Social work is one of the best disciplines for learning about and intervening in the phenomenon of disability in the migration process, as it is characterized by intervention in situations of social need and/or problems from which to promote the protection and assertion of social and human rights, paying special attention to those groups that are vulnerable and at risk of social exclusion. For this reason, the link between disability in the migratory process is pertinent as a challenge to be approached through professional practice, since it takes into account the necessary tools with which to favor social transformation [68].

Social work is a key consideration in the challenge to make visible, analyze and act on the reality of migrants who are disabled or who are affected by it due to their own displacement. It is the task of social intervention professionals to give a voice to these people, as well as to demand the response that their situation requires from governments and citizens [69].

Although immigration governance is increasingly Europeanized, the trend regarding integration governance directed at immigrants is more focused on the local level [70], since local policies are more sensitive and responsive to the needs of these groups than central policies. It is committed to proximity and a greater interrelation between the different actors involved in the process (local governments, public and private entities, immigrant associations, Non-Governmental Organizations (NGOs), etc.)

In short, the phenomenon of migration is not unidirectional; rather, there are many variables and processes that come into play and must be carefully considered. Therefore, intervention and the design of actions by social work and sociology professionals are necessary from a holistic perspective. These actions must be based on social diversity, superdiversity and respect for differences, and pave the way for public authorities, both nationally and internationally, to address the phenomenon of migrants and/or people with disabilities.

From the perspective of superdiversity, we must support action protocols that, while overcoming exclusion in a context of obvious inequalities, allow for the full inclusion of migrants in the hosting society, either from its applicability to social policy or from social innovation as a professional tool in achieving an inclusive society [71].

This review presents a summary of the responses that are being provided in Europe to the needs of migrants through the prism of superdiversity. In addition, the challenges facing agencies and institutions to improve the care given to refugees in host countries are listed. In this way, aspects as important as guaranteeing complete medical coverage for migrants and refugees are emphasized. So far, the institutional response to this phenomenon has been described as suboptimal [72]. Furthermore, this study tries to provide answers to problems related to the Common European Asylum System. There are certain questions presented at the European level that we have attempted to answer, such as “who needs international protection?” If the Member State of first entry is to take primary responsibility for the asylum procedure, what are the legal obligations that Member States have towards asylum seekers and beneficiaries? [73].

The results of this research are of interest to the scientific community and to the rest of the population, since they synthesize those factors that need improvement to guarantee the human rights of migrants and refugees; more specifically, of migrants and refugees who exhibit some kind of functional diversity. Accordingly, we present the political proposals that are being implemented to guarantee protection, security, access to resources, basic rights and to enhance the capacities of local authorities. The role of education as a key tool for coexistence is reinforced and the design of action protocols for coexistence is encouraged. The implications of this study are based on the detection of the various future challenges faced by both migrants and refugees with disabilities, as well as on the political approach to this situation.

Several facets of the migration phenomenon in Europe require more research in the future. It is important that studies in the near future establish a complete statistical record that shows the magnitude of the migration phenomenon in Europe and that is capable of counting the number of migrants with disabilities who arrive from different countries of origin. More scientific evidence is required of the difficulties and challenges facing migrants, both in their countries of origin and destination, as well as of the causes that force migrants to leave their native country. It is appropriate to review in depth the protocols and policies undertaken by different countries to deal with migration from the paradigm of superdiversity.

For destination countries, it is important to offer a synthesis of political proposals that provide guidelines for the future of migration and disability. Accordingly, based on our study, the following political actions are recommended:

− Create an information system to register and identify needs that can be used to plan and advance the design of ad hoc public policies aimed at migrants with disabilities.
− Provide durable solutions that allow migrants with disabilities to be inserted into the different protection systems of the host countries.
− Craft strategic coordination plans between countries to provide assistance and protection for migrants with disabilities.
− Incorporate the participation of the migrant population with disabilities in policy decision-making spaces.
